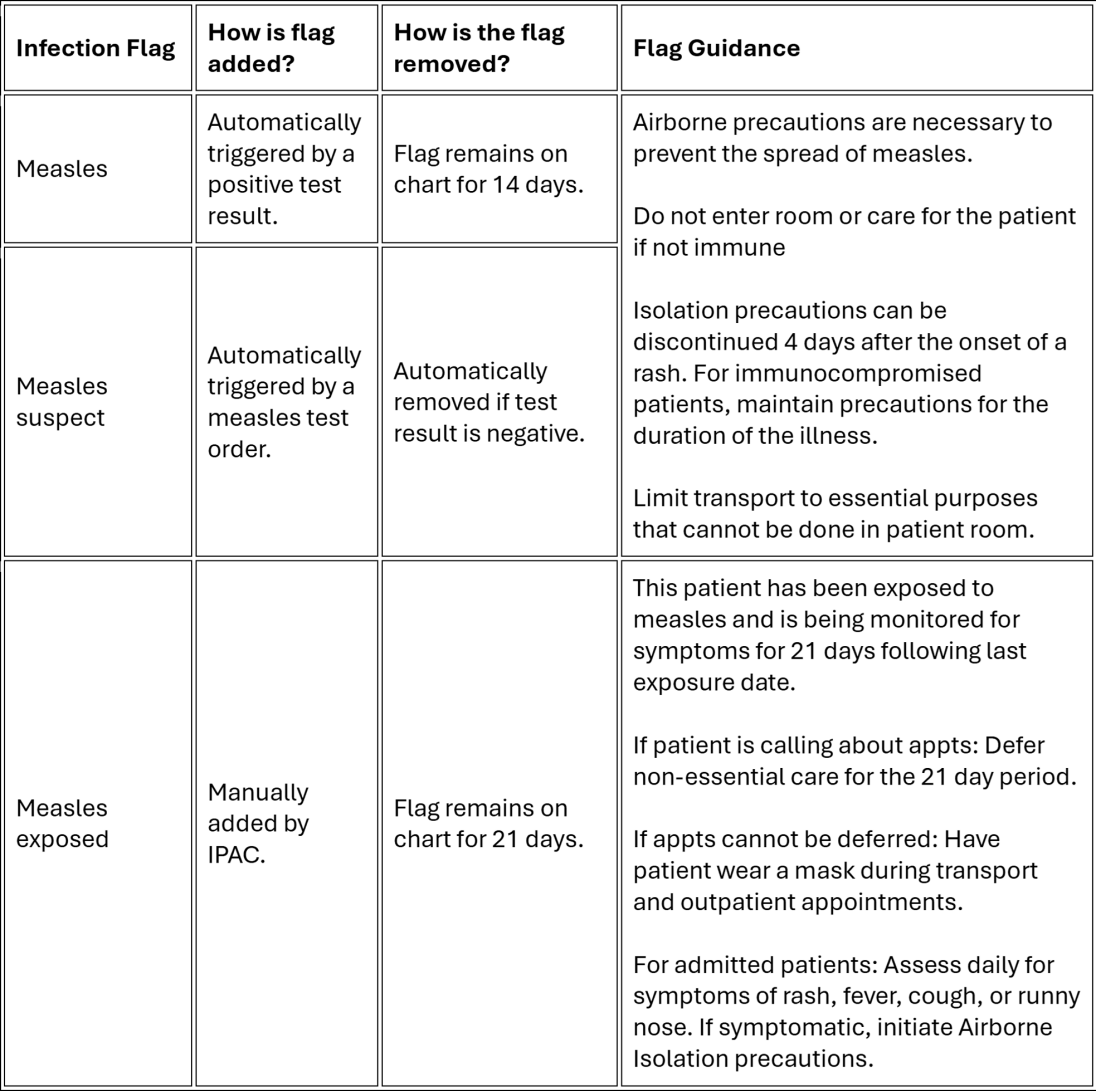# 334 Incorporating SARS-CoV-2 Case Surveillance Data in the Comparison of Wastewater Concentration Methods for Wastewater Surveillance

**DOI:** 10.1017/ash.2026.10678

**Published:** 2026-06-23

**Authors:** Jenna Rasmusson, Priya Sampathkumar, John OHoro, Jean Barth, Alyssa Olson, Ben Nelson, Rebecca Faller, Leah Siple

**Affiliations:** 1 Mayo Clinic; 2 Mayo Clinic, Rochester, MN; 3 Mayo Clinic College of Medicine

## Abstract

**Background:** In 2025, 50 measles outbreaks drove 87% of confirmed cases reported in the United States. As a highly infectious virus with an estimated R ranging from 12-18, we sought rapidly scalable and implementable measles preparedness measures for our facilities. **Methods:** The electronic health record (EHR) has flags that can be placed on patient records to denote the presence of, or concern for, active, transmissible infections. These flags are highly visible in the EHR and represent a good usability heuristic as front line providers are already trained to look for this information. Infection Prevention and Control (IPAC) worked with nursing informatics to develop measles-specific flags for suspected, confirmed and exposed patients in the EHR, expanding flagging functionality in preparation for potential measles cases and exposures. The flagging was tied to registry functionality to allow for tracking of exposed and confirmed cases longitudinally for supporting IPAC investigations in addition to clinical operations. **Results:** Three different flags were developed in the EHR to address measles. Specific criteria were created for each flag, as shown in Table 1. Table 1: Attached as Image **Conclusion:** As a highly contagious virus, measles presents unique challenges to healthcare facilities managing both cases and exposures. Measles flags within the EHR can be leveraged to assist facilities with case tracking, exposure management, and infection prevention and control.

**Figure:**